# Supplementary material for: The Impact of Omega-3 Fatty Acids on the Evolution of Acinetobacter baumannii Drug Resistance
Source: Microbiol Spectr. 2021 Nov 17;9(3):e01455-21. doi: 10.1128/Spectrum.01455-21 (PMC8585491; doi:10.1128/Spectrum.01455-21)
Supplement: SUPPLEMENTAL FILE 1 — Supplemental material. Download SPECTRUM01455-21_Supp_1_seq6.pdf, PDF file, 0.5 MB [file spectrum01455-21_supp_1_seq6.pdf]

The impact of omega-3 fatty acids on the evolution of *Acinetobacter baumannii* drug resistance

Maoge Zang[1], Felise G. Adams[1], Karl A. Hassan[2], Bart A. Eijkelkamp[1*]

[1]Molecular Sciences and Technology, College of Science and Engineering, Flinders University, Adelaide, South Australia, 5042, Australia

[2]School of Environmental and Life Sciences, University of Newcastle, Callaghan, New South Wales, 2308, Australia.

Running Title: Resistance development in *A. baumannii*.

Keywords: Antimicrobial host lipids, free fatty acids, macrolides, resistance evolution, RND efflux, AdeABC, AdeIJK.

* **Corresponding author:** Molecular Sciences & Technology, College of Science and Engineering, Flinders University, Adelaide, South Australia, 5042, Australia. Phone: +61-8-8201-7779. E-mail: bart.eijkelkamp@flinders.edu.au.

Supplementary Material

Supplementary tables

Table S1. Bacterial strains included in the study

Strain	Genotype/description	Reference/Source
AB5075_UW	Wild Type	Manoil lab (1)
adeN::*ISAba13*	AB5075_UW with integration of IS*Aba13* in intergenic region upstream of *adeN*	This study
adeI::ISAba1	AB5075_UW with integration of IS*Aba1* in coding region of *pgpB* (ABUW_0845)	This study
AdeS$_{D167N}$	AB5075_UW 1974323 C-T	This study
AdeS$_{F170L}$	AB5075_UW 1974312 A-T	This study
AdeS$_{P154S}$	AB5075_UW 1974362 G-A	This study
AdeR$_{I27S}$	AB5075_UW 2003503 A-C	This study
AB5075_A22	AB5075_UW from microevolution screen	This study

(1) Gallagher LA, Ramage E, Weiss EJ, Radey M, Hayden HS, Held KG, Huse HK, Zurawski DV, Brittnacher MJ, Manoil C. 2015. Resources for genetic and genomic analysis of emerging pathogen *Acinetobacter baumannii*. J Bacteriol 197:2027-35.

Table S2. Oligonucleotides used study

Gene	Forward (5’ - 3’)	Reverse (5’ - 3’)
adeB	GTCATGGGTTCAAGCGGTC	TTCACCCGATGACGTATCG
adeG	GCTTAAACCCAAGGGAGGC	TTGCCCTGTTAAGCCTGTC
adeJ	ATACGCAATGCGTATCTGG	ACTTGACCTTGTACAGCCG
adeN	TTTTTGAACCACCAGCATGA	TCCTTGAGTCACATCATCTC
GAPDH	CACCGTCGTACACGTGTTGT	CAACATCACCGCCTTTTTCT

Supplementary figures

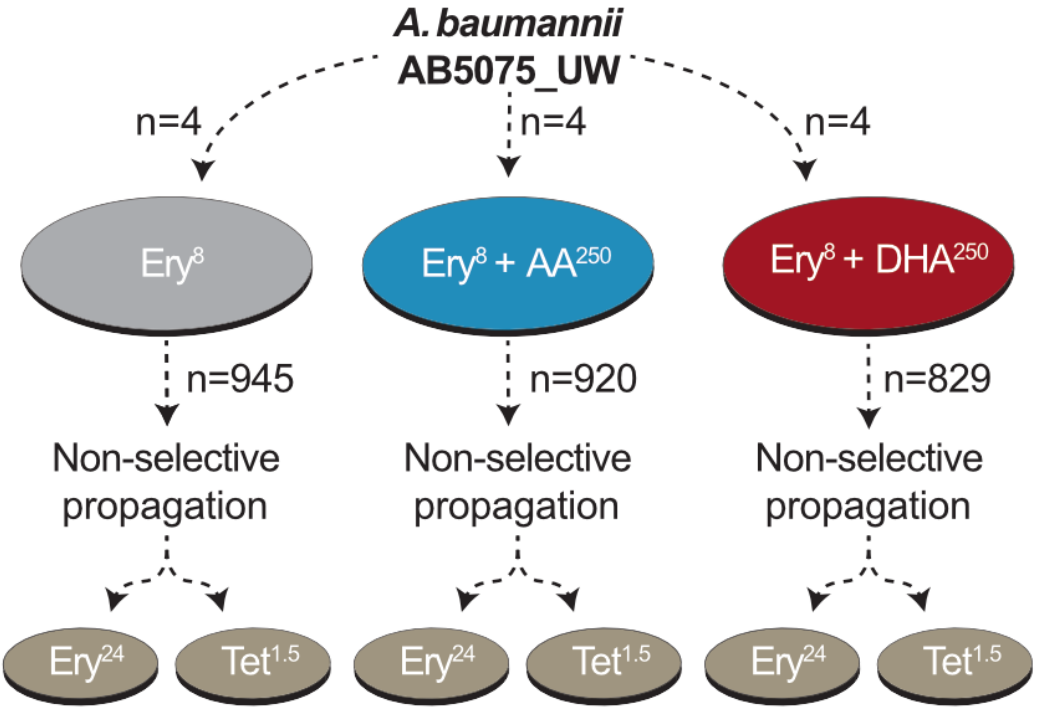

Figure S1. *A. baumannii* AB5075_UW microevolution experiments. Overnight cultures of *A. baumannii* were diluted and spread plated for single colonies on solid LB media containing 8 µg.ml^{-1} erythromycin ± 250 µM AA or DHA for culturing at 37°C for 24 hours before colonies were selected for propagation in LB media alone. The clone libraries were screened for a gain in erythromycin or tetracycline resistance by spotting onto LB media at 24 µg.ml^{-1} or 1.5 µg.ml^{-1} of antibiotic, respectively. The clone libraries were generated from 4 independent experiments.

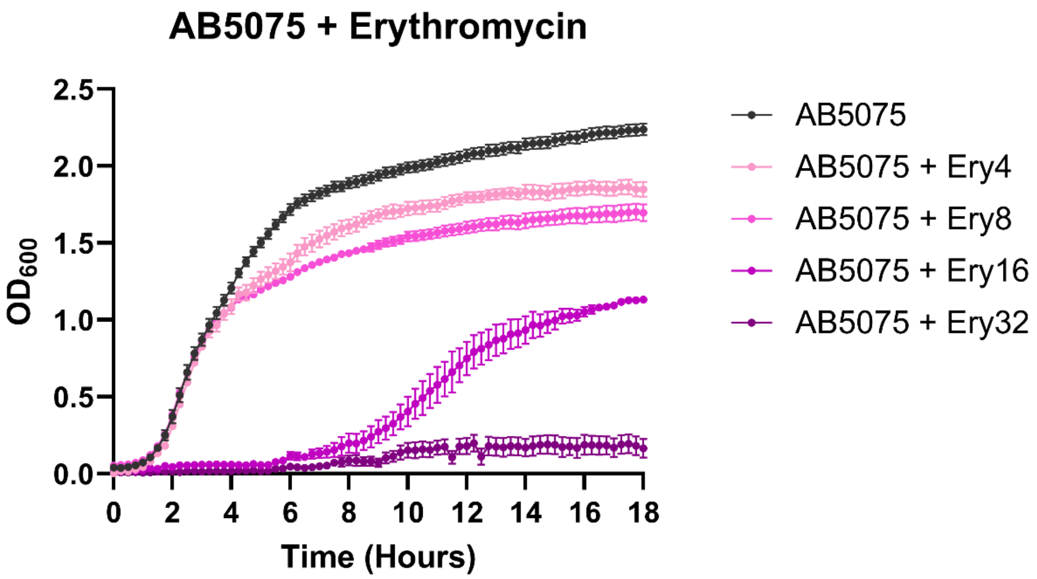

Figure S2. Impact of erythromycin on the growth of AB5075_UW. Growth inhibition of various concentrations of erythromycin (4, 8, 16, 32 µg.ml^{-1}) were examined. All data are the mean of at least biological triplicates (± SEM).

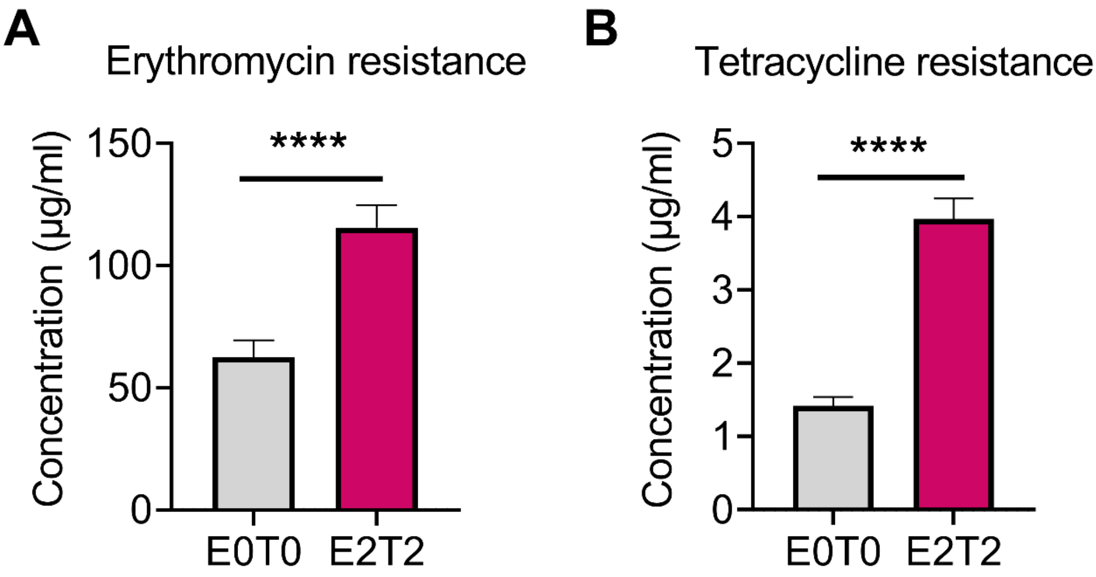

C

Strain	Ery MIC (µg.ml^{-1})	Tet MIC (µg.ml^{-1})
**AB5075_A22**	**64**	**1.25**
**AdeS$_{P154S}$**	**128**	**5**
**AdeS$_{D167N}$**	**128**	**5**
**AdeS$_{F170L}$**	**128**	**5**
**AdeR$_{I27S}$**	**128**	**5**
adeN::ISAba13	**64**	**5**
adeI::ISAba1	**64**	**5**

Figure S3. Antimicrobial resistance in the E0T0 and E2T2 clones. The minimal inhibitory concentration (MIC) of 22 E0T0 and 23 E2T2 clones to **(A)** erythromycin and **(B)** tetracycline was examined by 2-fold microdilution analyses. **(C)** The MICs of sequenced E2T2 clones are shown. All data are the mean of biological triplicates (± SEM). Statistical analyses were performed using a student's *t*-test (**** = p<0.0001).

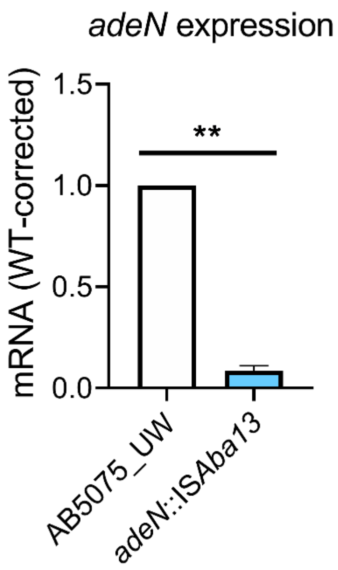

Figure S4. Expression of *adeN* in the *adeN*::IS*Aba13* mutant. The mRNA expression levels of *adeN* in the wild-type (AB5075_UW) and the E2T2 clone *adeN*::IS*Aba13* cells were examined using qRT-PCR. All data are the mean of biological triplicates (± SEM). Statistical analyses were performed using a student's *t*-test (** = $p<0.01$).
